# Postoperative Adjuvant Radiotherapy Can Delay the Recurrence of Desmoid Tumors After R0 Resection in Certain Subgroups

**DOI:** 10.3389/fsurg.2021.697793

**Published:** 2021-09-13

**Authors:** Tielong Yang, Haotian Liu, Zhichao Liao, Chao Zhang, Lijie Xiang, Jilong Yang

**Affiliations:** ^1^Department of Bone and Soft Tissue Tumor, Tianjin Medical University Cancer Institute and Hospital, Tianjin, China; ^2^National Clinical Research Center for Cancer (NCRCC), Key Laboratory of Cancer Prevention and Therapy, Tianjin's Clinical Research Center for Cancer, Tianjin Medical University Cancer Institute & Hospital, Tianjin, China

**Keywords:** desmoid tumor, adjuvant radiotherapy, aggressive surgery, recurrence, subgroup

## Abstract

**Background:** When patients with desmoid tumors (DTs) present uncontrolled clinical symptoms, surgery is an effective treatment, but the high postoperative recurrence rate is a major problem. The significance of adjuvant radiotherapy has been debated for many years, and the significance of aggressive surgery has not been reported.

**Methods:** Medical records for DT patients were collected. KM analysis and the Mann–Whitney *U*-test were performed to evaluate the role of radiotherapy and aggressive surgery in the entire cohort and different subgroups.

**Results:** Of 385 DT patients, 267 patients with R0 resection were included in the final analysis. A total of 53 patients (19.85%) experienced recurrence. Although radiotherapy showed no significant effect on recurrence-free survival (RFS) or time to recurrence (TTR) in the entire cohort, radiotherapy delayed recurrence in the age ≤ 30 years old subgroup (TTR = 35 months with surgery plus radiotherapy, TTR = 11 months with surgery alone; *p* = 0.014) and the tumor diameter >5 cm subgroup (TTR = 26 months with surgery plus radiotherapy, TTR = 11 months with surgery alone; *p* = 0.02) among patients with a single tumor. Aggressive surgery improved RFS in the tumor diameter >5 cm subgroup (*p* = 0.049) but not the entire cohort.

**Conclusions:** Although radiotherapy cannot improve RFS, it can delay recurrence in the age ≤ 30 years old subgroup and the tumor diameter >5 cm subgroup among patients with a single tumor. For patients with large invasive tumors and multiple involved sites, aggressive surgery could be selected to achieve complete tumor resection to improve RFS.

## Introduction

Desmoid tumors (DTs) are generally regarded as borderline or low-grade malignant tumors. Although they have no metastatic potential, they are prone to recurrence after surgery and may cause local damage (1). As a rare tumor, DT only accounts for 3% of all soft tissue tumors, and the incidence rate is only 5–6 per million. The incidence is twice as high in females as in males, and the onset peak is 30–40 years old (1). DT can be divided into two categories, namely, sporadic DT and familial adenomatous polyposis (FAP)-related DT. Mutation of the CTNNB1 gene encoding β-catenin in ~85–90% of sporadic DTs may lead to the accumulation of β-catenin in the nucleus, which may be related to the pathogenesis of DT (2). The three most common types of β-catenin mutations are T41A, S45F, and S45P, accounting for 50, 25, and 9%, respectively, of all mutations (3–5). FAP-related DTs are associated with APC gene mutations. CTNNB1 gene mutations and APC gene mutations are usually exclusive (3). Approximately 5–30% of FAP patients will develop DT, and the risk of DT in FAP patients is 1,000 times higher than that in the general population (6).

It is difficult to predict the natural course of DT, which presents great challenges in its treatment and prognosis assessment. A recent report showed that MRI is an important imaging method for evaluating the disease and prognosis (7). With a better understanding of the natural course of DT, the treatment of DT has dramatically changed. According to the latest DT management guidelines, close observation has become the preferred treatment for patients with DT. Surgical treatment should only be considered if clinical symptoms of pain or compression are present (2, 8, 9). However, the high postoperative recurrence rate has become a major problem affecting the prognosis. The majority of studies have reported that the postoperative recurrence rate exceeds 20%, and some reports even suggest that it exceeds 70% (10–13). Therefore, strategies for reducing postoperative recurrence are urgently needed.

Whether postoperative adjuvant radiotherapy can reduce the recurrence rate and improve the prognosis has been debated for decades. To date, there have been no prospective clinical trials to assess whether postoperative adjuvant radiotherapy can reduce recurrence, and studies assessing the effect of adjuvant radiotherapy are all retrospective in nature. Most of these studies involved only a few dozen or a hundred patients and came to divergent conclusions. Two studies involving more than 400 patients showed that although adjuvant radiotherapy did not reduce recurrence in all patients, it did so in the extremity tumor subgroup (11, 12). For the Chinese population, the vast majority of reports indicate that postoperative adjuvant radiotherapy cannot reduce recurrence; however, one study supposed that radiotherapy can reduce the recurrence rate of neck desmoid tumors (14–18). On the other hand, the role of aggressive surgery, such as multi-site/organ/tissue resection, has not been reported before. In the present study, we attempted to evaluate the efficacy of postoperative radiotherapy and aggressive surgery with R0 resection in DTs. Our data show that although radiotherapy cannot improve recurrence-free survival (RFS), it can delay recurrence in the age ≤ 30 years old subgroup and tumor diameter > 5 cm subgroup among patients with a single tumor. Aggressive surgery did not improve RFS in the entire cohort but improved RFS in the tumor diameter > 5 cm subgroup.

## Methods

### Data Collection

**Figure 1:**
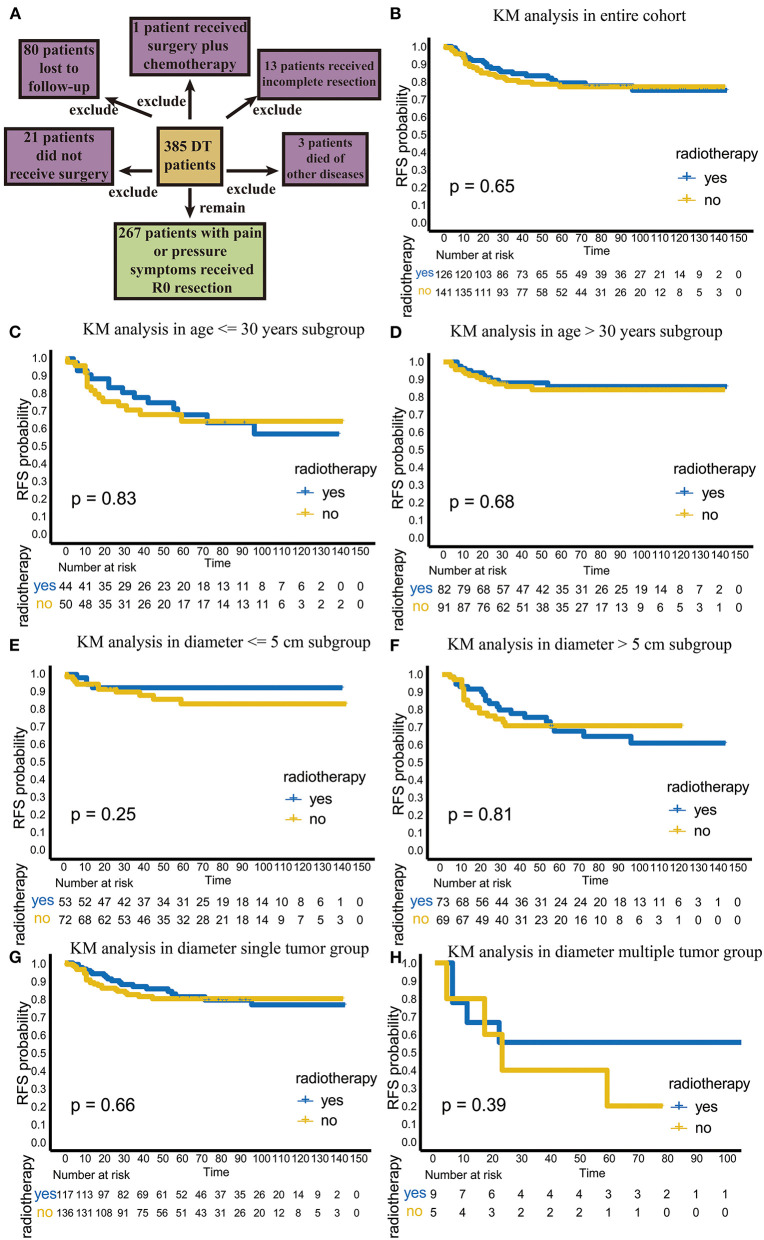
The flowchart of patient selection and efficacy analysis of radiotherapy in the entire cohort and different subgroups. **(A)** The flowchart of patients selection. **(B)** The efficacy of radiotherapy in entire cohort. **(C)** The efficacy of radiotherapy in the age ≤ 30 years old subgroup. **(D)** The efficacy of radiotherapy in the age > 30 years old subgroup. **(E)** The efficacy of radiotherapy in the diameter ≤ 5 cm subgroup. **(F)** The efficacy of radiotherapy in the >5 cm diameter subgroup. **(G)** The efficacy of radiotherapy in the single-tumor subgroup. **(H)** The efficacy of radiotherapy in the multiple-tumor subgroup.

With the approval of the ethics committee, we retrospectively and continuously collected the medical records of 385 patients with DT who were admitted to Tianjin Medical University Cancer Institute and Hospital from January 2007 to June 2019. To exclude the interference of other factors and obtain a homogeneous patient cohort, we excluded the following patients: (1) patients who were lost to follow-up, (2) patients who did not undergo surgery, (3) patients who died of other diseases, (4) patients receiving adjuvant chemotherapy after surgery, and (5) patients with incomplete resection (R1 and R2). The remaining 267 patients who showed uncontrolled clinical symptoms such as pain or compression and were treated with R0 resection surgery were included in the final analysis (Figure 1A). After carefully reviewing the medical records of these patients, we confirmed that they all underwent surgical resection because of symptoms of pain or compression, and the margin status was R0. The following variables were retrieved from patient records: age, sex, admission status, tumor location, tumor number, tumor diameter, radiotherapy status and recurrence status. Adjuvant radiotherapy was performed in patients who were considered to have a high probability of postoperative recurrence by a surgeon and radiation oncologist after the patients signed informed consent forms. According to our treatment experience and relevant guidelines, postoperative adjuvant radiotherapy for DT patients is mainly applied in the following patients: (1) patients with recurrent tumors; (2) patients with tumors located in the extremities, head and neck, or chest wall; and (3) patients with large tumor diameters (9, 19). Patients with two or more characteristics were more likely to receive adjuvant radiotherapy after surgery. On the other hand, for patients who have one of the above characteristics and are unwilling to receive adjuvant radiotherapy after being fully informed of the adverse effects of radiotherapy, only surgery can be performed. The radiotherapy dose varies from 50 to 60 Gy in fractions of 1.5–2.5 Gy.

### Data Analysis

Recurrence was the primary endpoint of this study and was defined as the appearance of a visible lesion or a lesion that can be assessed radiologically. RFS was defined as the time from surgery to recurrence (event) or the duration of follow-up (censoring). Time to recurrence (TTR) was defined as the time from surgery to recurrence only in patients who had recurrence. Univariate and multivariate Cox analysis was performed to screen recurrence-related risk factors. The relationship between radiotherapy and RFS in different subgroups was explored through Kaplan–Meier (KM) analysis, and comparisons between the subgroups were made with the log-rank test. The Mann–Whitney test was used to compare the difference in TTR in different subgroups. *P* < 0.05 was considered statistically significant, and all analyses were performed with IBM SPSS 22.

## Results

### Patient Characteristics

The 267 patient demographics and tumor characteristics are shown in Table 1. A total of 126 patients (47.2%) underwent radiotherapy after surgery, and 141 patients (52.8%) only received surgery. The ratio of male to female patients was ~1:2.3. In terms of tumor number, 253 patients (94.8%) had a single tumor, while only 14 patients (5.2%) had multiple tumors.

**Table 1:** The patients demographics and tumor characteristics.

	**Overall**	**Surgery plus radiotherapy**	**Only surgery**	***P*** **-value**
**Patients**	267	126 (47.2%)	141 (52.8%)	
**Admission status (%)**				0.001^***^
Primary	207 (77.5)	86 (68.3)	121 (85.8)	
Recurrent	60 (22.5)	40 (31.7)	20 (14.2)	
**Gender (%)**				0.16
Male	80 (30.0)	32 (25.4)	48 (34.0)	
Female	187 (70.0)	94 (74.6)	93 (66.0)	
**Age**				0.926
**Median [range]**	35.00 [1, 87]	36.00 [7,69]	35 [1,87]	
≤ 30 years	94 (35.2)	44 (34.9)	50 (35.5)	
>30 years	173 (64.8)	82 (65.1)	91 (64.5)	
**Site (%)**				0.332
Extremity	101 (37.8)	52 (41.3)	49 (34.8)	
Non-extremity	166 (62.2)	74 (58.7)	92 (65.2)	
**Disease number (%)**				0.298
Single	253 (94.8)	117 (92.9)	136 (96.5)	
Multiple	14 (5.2)	9 (7.1)	5 (3.5)	
**Diameter**				0.178
**Median [range]**	6.00 [1.00,20.00]	6.00 [1.50,20.00]	5.00 [1.00,20.00]	
≤ 5 cm	125 (46.8)	53 (42.1)	72 (51.1)	
>5 cm	142 (53.2)	73 (57.9)	69 (48.9)	

*** *p < 0.001*.

Aggressive surgery, such as multi-site/organ/tissue resection, was performed in 52 patients (19.5%), and the resection sites included local nerves, part of the ribs, part of the femur, part of the ilium, part of the lung tissue, part of the small intestine and part of the ileum, etc.

### Screening of Risk Factors for Recurrence

In the univariate Cox analysis, age, tumor diameter, admission status, location, and tumor number were associated with recurrence, and the recurrence rate was higher in patients ≤ 30 years old (HR: 2.505; *p* = 0.001), tumor diameter > 5 cm (HR: 2.837; *p* = 0.001), recurrent tumors (HR: 2.648; *p* < 0.001), extremity tumors (HR: 1.800; *p* = 0.033), and multiple tumors (HR: 3.960; 95% CI: 1.865–8.408, *p* < 0.001; Table 2).

**Table 2:** Univariate and multivariate Cox analysis of risk factors associated with recurrence.

**Factor**	**Univariate**		**Multivariate**	
	**HR (95%CI)**	***P*** **-value**	**HR (95%CI)**	***P*** **-value**
**Gender**				
Male vs. female	1.054 (0.592–1.878)	0.858		
**Age**				
≤ 30 vs. >30 years	2.505 (1.455–4.314)	0.001^***^	2.145 (1.234–3.730)	0.007^**^
**Diameter**				
>5 vs. ≤ 5 cm	2.837 (1.538–5.232)	0.001^***^	2.717 (1.449–5.095)	0.002^**^
**Admission status**				
Recurrent vs. primary	2.648 (1.533–4.575)	<0.001^***^	1.678 (0.948–2.970)	0.076
**Location**				
Extremity vs. non-extremity	1.800 (1.048–3.091)	0.033^*^	1.279 (0.715–2.290)	0.407
**Tumor number**				
Multiple vs. single	3.960 (1.865–8.408)	<0.001^***^	3.918 (1.728–8.882)	0.001^***^
**Aggressive surgery**				
Yes vs. no	0.754 (0.355–1.601)	0.463		
**Radiotherapy**				
Yes vs. no	0.882 (0.514–1.516)	0.651		

* *p < 0.05,*

** *p < 0.01, and*

*** *p < 0.001. Bold values indicate the name of the variable or the p value of the variable that makes sense*.

In the multivariate Cox analysis, only age, tumor diameter and tumor number were associated with recurrence, and the recurrence rate was higher in patients ≤ 30 years old (HR: 2.145; *p* = 0.007) and those with tumor diameter > 5 cm (HR: 2.717; *p* = 0.002) and multiple tumors (HR: 3.918; *p* = 0.001; Table 2).

Then, we explored the efficacy of radiotherapy in the entire cohort and in three independent risk factor subgroups.

### Adjuvant Radiotherapy Showed No Effect on RFS and TTR in the Entire Cohort

Among the 267 patients, 126 received postoperative adjuvant radiotherapy. Among these patients 24 (19%) had recurrence. A total of 141 patients received surgical treatment only, and 29 of these patients (20.6%) had recurrence. There was no significant difference in the recurrence rate between these two groups (*p* = 0.756, χ^2^ = 0.97). In the KM analysis, we also found that radiotherapy did not improve RFS or local control in the total population (*p* = 0.65; Figure 1B).

Next, we further compared the effects of radiotherapy on TTR. The results showed that in the entire cohort, the TTR of patients undergoing surgery plus radiotherapy was 21.5 (range, 4–96) months, while the TTR of patients undergoing surgery alone was 13 (range, 1–59) months (*p* = 0.128), indicating that radiotherapy could neither significantly reduce recurrence nor significantly delay recurrence in the total population, even though the adjuvant radiotherapy group had a longer TTR (Table 3).

**Table 3:** TTR comparison between surgery plus radiotherapy and only surgery.

**Group (median [range])**	**Surgery plus radiotherapy**	**Only surgery**	***p*** **-value**
**Entire cohort**	21.5 (4–96)	13 (1–59)	*p* = 0.128
**Age**			
≤ 30 years	25.5 (4–96)	12 (1–59)	*p* = 0.139
>30 years	17 (7–53)	13 (4–45)	*p* = 0.475
**Disease number**			
Single	23 (4–96)	11 (1–45)	***p*** = 0.028^*^
Multiple	8.5 (6–22)	20 (4–59)	*p* = 0.384
**Diameter**			
≤ 5 cm	11 (6–14)	17 (1–59)	*p* = 0.523
>5 cm	23 (4–96)	11 (4–32)	***p*** **=** 0.043^*^
**Location**			
Trunk	24 (4–96)	12 (5–38)	*p* = 0.405
Extremity	20.5 (6–42)	23 (1–59)	*p* = 0.845

* *p < 0.05. Bold values indicate the name of the variable or the p value of the variable that makes sense*.

### Adjuvant Radiotherapy Showed No Effect on RFS and TTR in Different Age Subgroups

We further analyzed the efficacy of radiotherapy in different subgroups. First, we explored recurrence outcomes in the different age groups. In the age ≤ 30 years old subgroup, radiotherapy was not significant statistically (*p* = 0.83; Figure 1C); the TTR was 25.5 (range, 4–96) months for patients undergoing surgery plus radiotherapy, while the TTR was 12 (1–59) months for patients undergoing surgery alone (*p* = 0.139; Table 3). Similar results were found in patients over 30 years old (Figure 1D; Table 3). This result suggests that adjuvant radiotherapy does not affect RFS and TTR in different age subgroups.

### Adjuvant Radiotherapy Delayed Recurrence in the Tumor Diameter > 5 cm Subgroup

Next, we analyzed the efficacy of radiotherapy in different tumor diameter subgroups. Radiotherapy did not improve RFS in the tumor diameter ≤ 5 cm subgroup (*p* = 0.25) (Figure 1E). The TTR was 11 (range, 6–14) months for patients receiving surgery plus radiotherapy and 17 (range, 1–59) months for patients receiving surgery alone (*p* = 0.523; Table 3). However, in the tumor diameter > 5 cm subgroup, although radiotherapy did not improve RFS (*p* = 0.81; Figure 1F), the TTR of patients receiving surgery plus radiotherapy was significantly longer than that of patients receiving surgery alone (23 months for surgery plus radiotherapy group, 11 months for only surgery group, *p* = 0.043; Table 3). This suggests that radiotherapy can delay recurrence in the tumor diameter > 5 cm subgroup.

### Adjuvant Radiotherapy Could Delay Recurrence in a Single-Tumor Subgroup

Third, we explored the effect of radiotherapy on RFS in single- or multiple-tumor groups. Radiotherapy did not improve RFS in the single-tumor subgroup (*p* = 0.66; Figure 1G). The TTR was 23 (range, 4–96) months for patients receiving surgery plus radiotherapy and 11 (range, 1–45) months for patients receiving surgery alone (*p* = 0.028), which suggests that radiotherapy can delay recurrence in the single-tumor subgroup (Table 3). In the multiple-tumor subgroup, radiotherapy did not reduce recurrence (*p* = 0.39; Figure 1H) or delay recurrence (*p* = 0.384; Figure 1F; Table 3).

### Adjuvant Radiotherapy Could Delay Recurrence in the Tumor Diameter > 5 cm Subgroup and Age ≤ 30 Years Subgroup of Patients With a Single Tumor

As seen from the above analysis, adjuvant radiotherapy has a tendency to delay recurrence in patients with a single tumor but has a tendency to accelerate recurrence in patients with multiple tumors. For this reason, we believe that for the total patient population of 267 patients, although radiotherapy could delay recurrence in the tumor diameter > 5 cm subgroup (*p* = 0.043), the effect was actually caused by single tumor subgroup. It is worth considering that for DTs, multiple tumors are very rare. In our study cohort, only 5.2% of the patients had multiple tumors. Therefore, we limited the patient scope to 253 patients with a single tumor and performed a comparison of TTR between the two groups as shown in Table 4.

**Table 4:** TTR comparison between surgery plus radiotherapy and only surgery in single tumor group.

**Group (median [range])**	**Surgery plus radiotherapy**	**Only surgery**	***p*** **-value**
**Single tumor group**	23 (4–96)	11 (1–96)	***p*** = 0.028^*^
**Age**			
>30 years	20 (7–53)	12 (4–45)	*p* = 0.462
**Diameter**			
≤ 5 cm	12.5 (11–14)	17 (1–45)	*p* = 0.77
>5 cm	26 (4–96)	11 (4–32)	***p*** = 0.02^*^
**Location**			
Trunk	28 (4–96)	11 (5–38)	*p* = 0.174
Extremity	20.5 (11–42)	18 (1–45)	*p* = 0.734

* *p < 0.05. Bold values indicate the name of the variable or the p value of the variable that makes sense*.

In the tumor diameter > 5 cm subgroup, the TTR was 26 (range, 4–96) months for patients receiving surgery plus radiotherapy and 11 (range, 4–32) months for patients receiving surgery alone (*p* = 0.02; Table 4). Our results suggests that radiotherapy could delay recurrence in the tumor diameter > 5 cm subgroup of patients with a single tumor.

In the age ≤ 30 years subgroup, the TTR was 35 (range, 4–96) months for patients receiving surgery plus radiotherapy and 11 (range, 1–38) months for patients receiving surgery alone (*p* = 0.014; Table 4), which indicated that surgery plus radiotherapy could delay recurrence in both the tumor diameter > 5 cm subgroup and the age ≤ 30 years subgroup of patients with a single tumor.

### Aggressive Surgery Improved RFS in the Subgroup With Tumor Diameter > 5 cm

Among the 267 patients, 52 underwent aggressive surgery, such as multi-site/organ/tissue resection with or without reconstruction. In all, eight patients (15.4%) experienced recurrence. There were 215 patients who received only R0 tumor resection, and 45 patients (20.9%) had recurrence. Even though aggressive surgery decreased the recurrence rate, there was no significant difference in recurrence rate between these two groups (*p* = 0.368, χ^2^ = 0.809).

**Figure 2:**
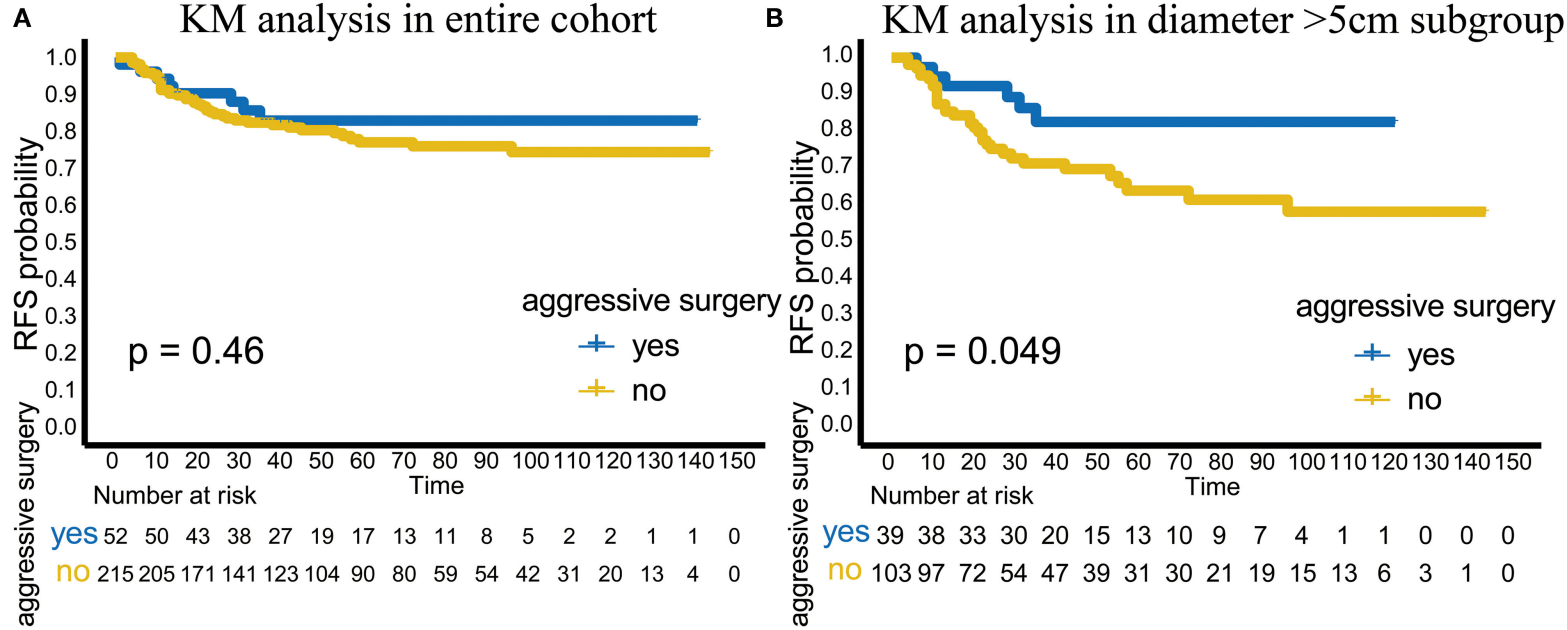
Analysis of aggressive surgery in different groups. **(A)** The efficacy of aggressive surgery in the entire cohort. **(B)** The efficacy of aggressive surgery in the tumor diameter >5 cm subgroup.

In terms of RFS, aggressive surgery, such as multi-site/organ/tissue resection, was not beneficial in improving RFS in the entire cohort (*p* = 0.46; Figure 2A). However, in the subgroup with tumor diameter > 5 cm, patients with aggressive surgery had better RFS (*p* = 0.049), suggesting that for patients with large invasive tumors, aggressive surgery could be selected to achieve complete tumor resection to improve RFS (Figure 2B).

**Figure 3:**
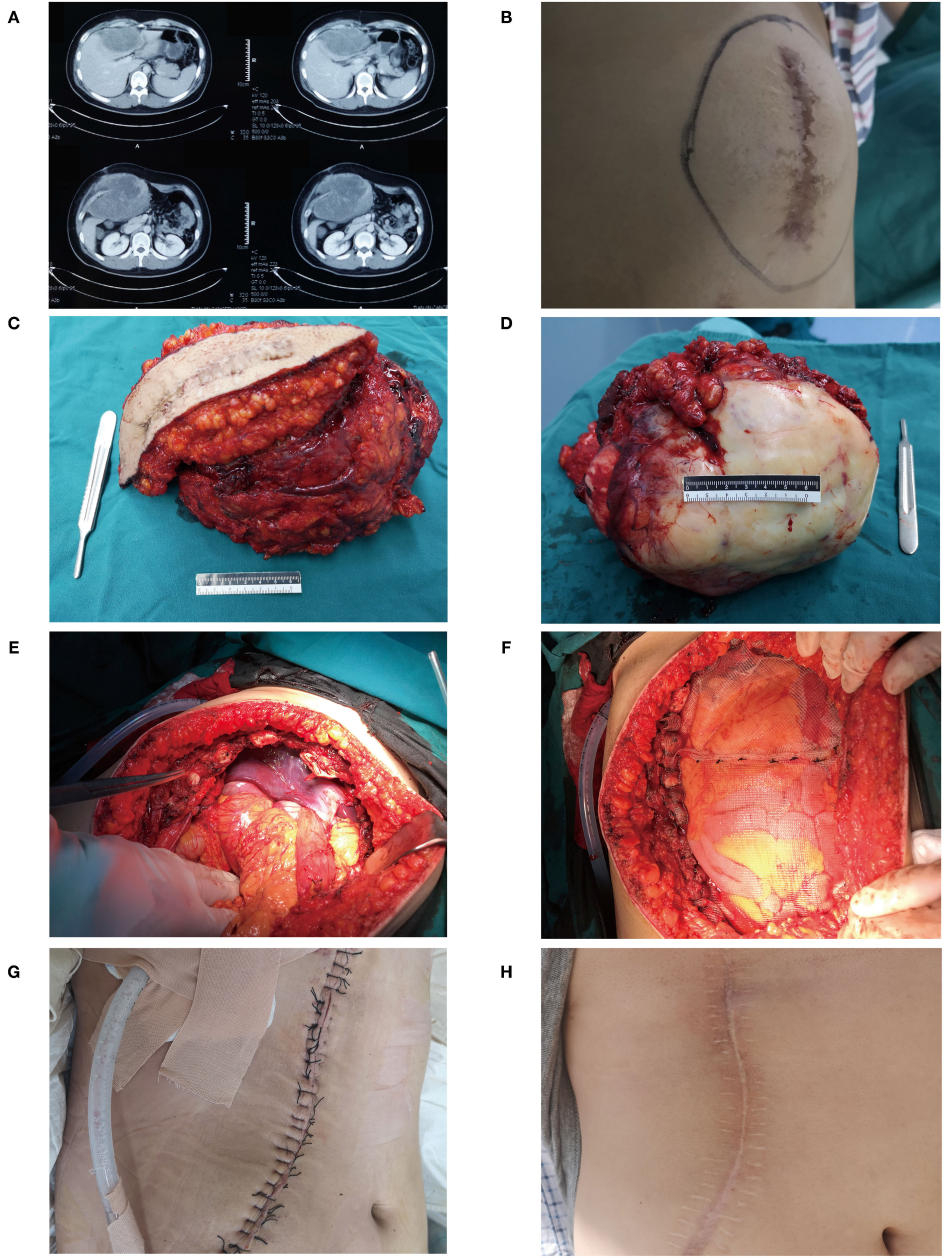
A typical case of DT in a 20-year-old male. **(A)** Preoperative CT scan showed a large mass in the right anterior abdominal wall, and the liver had low-density areas due to tumor compression. **(B)** A huge mass can be seen at the right chest wall. **(C)** The previous surgical scar was removed with the tumor. **(D)** A gross specimen of the tumor. **(E)** The patient underwent aggressive surgery, and the invaded diaphragm and ribs were removed. **(F,G)** We reconstructed the junction of the right chest wall and abdominal wall with a biological patch. **(H)** During the follow-up, the patient recovered well.

Aggressive surgery was seldom selected to treat DTs unless patients had serious clinical symptoms. For example, a 20-year-old male was referred to our hospital for postoperative recurrence of DT. A preoperative CT scan showed a large mass in the right anterior abdominal wall, which had invaded the entire abdominal wall and was growing into the abdominal cavity (Figure 3A). The liver was deformed under pressure due to tumor compression, and low-density areas appeared near the tumor (Figure 3A). There was a large mass at the junction of the right chest wall and abdominal wall with uncontrolled pain in the chest and abdominal walls (Figure 3B). Liver function was slightly impaired, and ALT and AST were increased to varying degrees, at 80 and 53 U/L, respectively. To prevent pain, aggressive surgery was prepared, and multiple disciplinary team (MDT) panel discussions including the hepatological surgery department and thoracic surgery department were performed before the operation. Aggressive surgery was administered with reconstruction of the right chest wall and abdominal wall. The tumor, including the old scar, was completely removed, and the tumor volume was 19 cm × 18 cm × 17 cm (Figures 3C,D). During the operation, the invaded diaphragm and ribs were removed (Figure 3E). After tumor resection, we reconstructed the junction of the right chest wall and abdominal wall with a biological patch (Figures 3F,G). During follow-up, the patient recovered well (Figure 3H).

## Discussion

Although the incidence and mortality of DT are very low, the high postoperative recurrence rate is still the most important obstacle in the treatment of DT; the significance of postoperative adjuvant radiotherapy is also controversial. In addition, the significance of aggressive surgery, such as multi-site/organ/tissue, has not been studied before. Therefore, in the present study, we explored the effect of radiotherapy and aggressive surgery on RFS in the entire cohort and different subgroups to determine the optimal treatment strategy for patients. With the largest Chinese DT cohort, our data suggest that some specific patients with R0 resection, such as the those in the age ≤ 30 years subgroup and tumor diameter > 5 cm subgroup of patients with a single tumor, might benefit from adjuvant radiotherapy. Additionally, for patients with large invasive tumors and multiple involved sites, aggressive surgery should be selected to achieve complete tumor resection to improve RFS.

The efficacy of postoperative adjuvant radiotherapy has been controversial for decades. Some studies have shown that adjuvant radiotherapy can reduce the recurrence rate, but other studies have shown that radiotherapy does not improve the prognosis (20–24). In addition, some studies have reported that the efficacy of radiotherapy is related to the margin status. A study involving 177 patients showed that in patients with positive margins, postoperative adjuvant radiotherapy improved the local control rate and reduced the recurrence rate compared to that with surgery alone (13). Another study that included 61 patients reached the same conclusion (25). Similarly, a meta-analysis of 1,295 patients from 16 studies showed that adjuvant radiotherapy was not beneficial for patients with negative margins but could improve local control and reduce recurrence in patients with incomplete resection (26). In the National Comprehensive Cancer Network (NCCN) guidelines, adjuvant radiotherapy is recommended for patients with R1 resection, and definitive radiotherapy is recommended for patients with R2 resection. However, because of the rarity of DTs, the number of patients included in these studies was small, mostly between a few dozen and a hundred. To date, only two retrospective studies analyzing the significance of postoperative adjuvant radiotherapy have included more than 400 patients. Crago et al. (12) suggested that only in the extremity tumor subgroup did adjuvant radiation improve local control (71 vs. 56%, *p* = 0.029) and reduce the recurrence rate (by 15%). Bishop et al. (11) also proposed that adjuvant radiotherapy can improve local control rates in the extremity tumor subgroup.

For the Chinese population, most of the studies reported that adjuvant radiotherapy does not reduce the recurrence rate (15–18). Among them, the study by Huang included 214 patients. However, a retrospective study involving 68 patients with neck desmoid tumors indicated that the 3-year event-free survival (EFS) of patients undergoing surgery plus radiotherapy was higher than that of patients receiving surgery alone (74.6 vs. 13.3%) (14).

As the largest retrospective study (*n* = 267 patients) in China to analyze the efficacy of adjuvant radiotherapy, our results are similar to those of Shin et al., who concluded that radiotherapy did not improve the long-term prognosis but could delay recurrence. However, Shin et al. (27) did not further discuss different subgroups. Our results showed that postoperative adjuvant radiotherapy could not improve RFS in the general population or any subgroup. However, in the age ≤ 30 years subgroup and tumor diameter > 5 cm subgroup of patients with a single tumor, adjuvant radiotherapy prolonged the postoperative recurrence time and delayed recurrence. In fact, for the general population, the TTR of surgery plus radiotherapy was 21.5 (4–96) months, while the TTR of patients undergoing surgery alone was 13 (1–59) months. Although the effect of radiotherapy was not statistically significant (*p* = 0.128), the trend and potential of adjuvant radiotherapy to delay recurrence could still be observed. We hypothesized that the multiple-tumor subgroup may mask this trend because in the multiple-tumor subgroup, the TTR of patients undergoing surgery plus radiotherapy was actually shorter than the TTR of patients undergoing surgery alone. When the analysis was restricted to the single-tumor subgroup, this trend became apparent. Considering that for DTs, patients with a single tumor account for the vast majority, this strategy is appropriate. We found that radiotherapy could delay recurrence in the age ≤ 30 years subgroup and the tumor diameter > 5 cm subgroup. Therefore, in future treatment, radiotherapy can be used as an adjuvant therapy to delay recurrence for patients with a single tumor and age ≤ 30 years or tumor diameter > 5 cm, but for patients with multiple tumors, adjuvant radiotherapy may actually accelerate recurrence.

As an intermediate tumor type with a low mortality rate, DT is characterized by a high recurrence rate. However, aggressive surgery is still not recommended, and active surveillance is the preferred treatment. However, aggressive surgery can sometimes be selected if the patients suffer from critical clinical symptoms caused by tumor pressure, invasion and organ involvement. In our cohort, although aggressive surgery, such as multi-site/organ/tissue resection, did not benefit the patients in the entire cohort (*p* = 0.46), the patients who received aggressive surgery had better RFS (*p* = 0.049) in the tumor diameter > 5 cm subgroup. Considering the effect of adjuvant radiotherapy on TTR described previously, our study showed that patients with tumor diameter > 5 cm are a special subgroup. In this special group, postoperative adjuvant radiotherapy could delay recurrence. Moreover, for patients with large invasive tumors and multiple involved sites, aggressive surgery could effectively improve RFS.

As a retrospective study, the present research has inherent limitation. First, the CTNNB1 gene mutation status was not included. Several studies have shown that the mutation type of the CTNNB1 gene is related to the probability of postoperative recurrence and that patients with S45F mutations have the highest postoperative recurrence rate among all patients (5, 10, 28). We tried to find the gross specimens of patients with these features in the specimen bank but could find only a few specimens, which was not enough to complete the subsequent analysis. In future work, we will pay attention to collecting gross specimens of DT patients to explore the relationship between CTNNB1 gene mutation types and radiotherapy. Second, information on recurrence was not obtained in three patients due to death of other causes, which may bias the final results.

Our findings have positive implications for guiding clinical treatment. For example, for patients with a single tumor and aged ≤ 30 years or with tumor diameter > 5 cm, postoperative adjuvant radiotherapy can be chosen because although radiotherapy cannot improve RFS, it can delay the recurrence, thus improving the quality of life in the early stage. Furthermore, if the tumor invades multiple sites, aggressive surgery, such as multi-site/organ/tissue resection, is recommended to improve RFS. On the other hand, for patients with a diameter ≤ 5 cm, aged >30 years old or with multiple tumors, adjuvant radiotherapy should not be included in the treatment plan because radiotherapy can neither reduce recurrence nor delay recurrence in these patients.

## Conclusions

In summary, we investigated the effect of adjuvant radiotherapy and aggressive surgery. Radiotherapy could delay recurrence in the age ≤ 30 years old subgroup and the tumor diameter > 5 cm subgroup among patients with a single tumor, and aggressive surgery could improve RFS in the tumor diameter > 5 cm subgroup, providing recommendations for the treatment of different populations based on data from our single institution. Large prospective clinical trials are still warranted to further explore and validate the effect of radiotherapy on postoperative patients with DTs.

## Data Availability Statement

The raw data supporting the conclusions of this article will be made available by the authors.

## Ethics Statement

All the procedures of this retrospective study were approved by the Institute Research Medical Ethics Committee of Tianjin Medical University Cancer Institute & Hospital ethics committee and performed according to the Helsinki declaration. All the patients signed a fully written, informed consent form at the time of admission; this form explained that the clinical data might be used for scientific research but would not compromise patient privacy.

## STROBE Statement

We declare that the manuscript conforms to the STROBE criteria.

## Author Contributions

TY and HL drafted the manuscript. ZL, CZ, LX, and JY revised the manuscript. All authors approved the final manuscript.

## Funding

This work was supported by the Key Nature Science Foundation of Tianjin (18YFZCSY00550 to JY).

## Conflict of Interest

The authors declare that the research was conducted in the absence of any commercial or financial relationships that could be construed as a potential conflict of interest.

## Publisher's Note

All claims expressed in this article are solely those of the authors and do not necessarily represent those of their affiliated organizations, or those of the publisher, the editors and the reviewers. Any product that may be evaluated in this article, or claim that may be made by its manufacturer, is not guaranteed or endorsed by the publisher.

## Abbreviations

DT: desmoid tumor
CI: confidence interval
HR: hazard ratio
RFS: recurrence free survival
TTR: time to recurrence
FAP: familial adenomatous polyposis.
